# Pyrolysis and co-pyrolysis of *Laminaria japonica* and polypropylene over mesoporous Al-SBA-15 catalyst

**DOI:** 10.1186/1556-276X-9-376

**Published:** 2014-08-01

**Authors:** Hyung Won Lee, Suek Joo Choi, Sung Hoon Park, Jong-Ki Jeon, Sang-Chul Jung, Sang Chai Kim, Young-Kwon Park

**Affiliations:** 1 Graduate School of Energy and Environmental System Engineering, University of Seoul, Seoul 130-743, South Korea; 2 Department of Environmental Engineering, Sunchon National University, Suncheon 540-950, South Korea; 3 Department of Chemical Engineering, Kongju National University, Cheonan 330-717, South Korea; 4 Department of Environmental Education, Mokpo National University, Muan 534-729, South Korea; 5 School of Environmental Engineering, University of Seoul, Seoul 130-743, South Korea

**Keywords:** Catalytic co-pyrolysis, *Laminaria japonica*, Polypropylene, Al-SBA-15

## Abstract

The catalytic co-pyrolysis of a seaweed biomass, *Laminaria japonica*, and a typical polymer material, polypropylene, was studied for the first time. A mesoporous material Al-SBA-15 was used as a catalyst. Pyrolysis experiments were conducted using a fixed-bed reactor and pyrolysis gas chromatography/mass spectrometry (Py-GC/MS). BET surface area, N_2_ adsorption-desorption isotherms, and NH_3_ temperature programmed desorption were measured to examine the catalyst characteristics. When only *L. japonica* was pyrolyzed, catalytic reforming slightly increased the gas yield and decreased the oil yield. The H_2_O content in bio-oil was increased by catalytic reforming from 42.03 to 50.32 wt% due to the dehydration reaction occurring on the acid sites inside the large pores of Al-SBA-15. Acids, oxygenates, mono-aromatics, poly aromatic hydrocarbons, and phenolics were the main components of the bio-oil obtained from the pyrolysis of *L. japonica*. Upon catalytic reforming over Al-SBA-15, the main oxygenate species 1,4-anhydro-*d*-galactitol and 1,5-anhydro-*d*-manitol were completely removed. When *L. japonica* was co-pyrolyzed with polypropylene, the H_2_O content in bio-oil was decreased dramatically (8.93 wt% in the case of catalytic co-pyrolysis), contributing to the improvement of the oil quality. A huge increase in the content of gasoline-range and diesel-range hydrocarbons in bio-oil was the most remarkable change that resulted from the co-pyrolysis with polypropylene, suggesting its potential as a transport fuel. The content of mono-aromatics with high economic value was also increased significantly by catalytic co-pyrolysis.

## Background

The development of renewable and sustainable energy resources is one of the most urgent tasks that scientists and engineers are facing owing to limited fossil fuel reserves and accelerating global warming. Compared to other renewable energies, such as solar energy, which require relatively long time for research and development, biomass is expected to be capable of replacing fossil fuels with much less efforts. Unlike crude oil, biomass is distributed evenly over the world and its quantity is gigantic, which makes biomass a promising energy source of the future.

Pyrolysis, which is a well-known method to produce energy from biomass, is a thermal conversion process producing a liquid fuel called bio-oil. The bio-oil produced from catalytic pyrolysis of biomass normally exhibit low oxygen content, high heating value, and improved miscibility with petroleum-derived liquid fuels.

While lignocellulosic biomass has widely been used as a feedstock for catalytic pyrolysis, macroalgae, including various seaweeds, are recently receiving significant attention as a new biomass material for energy production. The high photosynthetic efficiency of seaweeds, compared to that of woody land biomass, arouses an anticipation of producing bio-oil more effectively [1]. However, the pyrolysis bio-oil of seaweeds often displays severe instability, requiring catalytic reforming to improve the stability of the oil [1,2]. The research on the catalytic pyrolysis of macroalgae is still limited, compared to that for land biomass. Application of various catalysts to the pyrolysis of macroalgae needs to be investigated to realize the potential of macroalgae as an energy source.

Mesoporous catalysts can be good candidates for the catalytic pyrolysis of biomass because their large pore size is beneficial for the catalytic cracking of large-molecular-mass species during the pyrolysis process [3]. For instance, a mesoporous catalyst Al-SBA-15 was used in the catalytic pyrolysis of herb residue or miscanthus, leading to the production of valuable components such as phenolics [3,4].

Organic waste can also be used to produce energy. For example, a substantial amount of plastics are produced, consumed, and discarded. Waste plastics can be used to produce liquid fuel through pyrolysis. The pyrolysis oil produced from plastics is composed mostly of carbon and hydrogen, with only a limited content of oxygen, because plastics are produced from fossil fuels that contain much less oxygen than normal biomass materials. Therefore, if waste plastics are pyrolyzed together with biomass materials, they provide carbon and hydrogen and lower the oxygen content, resulting in an improvement of the oil quality [5]. This co-pyrolysis of biomass and plastics has recently been investigated actively [6-17]. However, the co-pyrolysis of macroalgae and plastics has never been studied yet.

In this study, a representative mesoporous catalyst Al-SBA-15 was applied to the catalytic pyrolysis of *Laminaria japonica*, a kind of seaweed, for the first time. The co-pyrolysis of polypropylene (PP), which is a representative plastic material, and *L. japonica* was also investigated for the first time.

## Methods

### *L. japonica* and PP

Proximate analyses of *L. japonica* and PP were conducted using a method suggested in a previous study [1,2]. *L. japonica* was shown to consist of moisture (7.7%), volatile matter (53.1%), fixed carbon (11.0%), and ash (28.3%) on a mass basis, whereas most mass (99.8%) was volatiles with only 0.2% of ash in the case of PP. Elemental analyses showed that *L. japonica* was composed of C (30.6%), H (4.9%), O (62.4%), N (1.5%), and S (0.5%) on a mass basis, whereas PP was composed only of C (85.4%) and H (14.6%).

### Synthesis and characterization of the catalyst

Mesoporous Al-SBA-15 was synthesized using a method suggested in a previous study [3]. The characterization of the synthesized catalyst was performed using BET, N_2_ adsorption-desorption analysis, X-ray diffraction patterns (XRD) and temperature-programmed desorption (TPD) of ammonia. Refer to a previously published report for more detailed analysis procedure [1,3].

### Catalytic pyrolysis and co-pyrolysis using a fixed-bed reactor

A U-type quartz reactor was used to investigate the change in the yields of gas and bio-oil by co-pyrolysis. To make an oxygen-free condition, 50-mL/min nitrogen gas flow was used to purge the reactor for 30 min prior to each experiment. Experiments were conducted with a 5-g *L. japonica* sample for 1 h at 500°C using 50-mL/min N_2_ gas as the carrier gas. In the case of co-pyrolysis of *L. japonica* and waste plastics, a mixture of 2.5-g *L. japonica* and 2.5-g PP was used for the experiments. In the case of catalytic pyrolysis, a catalyst/feedstock ratio of 1/10 was used. The pyrolysis product oil was collected in two consecutive condensers maintained at −20°C. A Teflon bag (DuPont Co., Wilmington, DE, USA) was installed after the condensers to collect the gaseous species that were not condensed in the condensers owing to their too low boiling points. The H_2_O content in bio-oil was analyzed using a Karl Fischer Titrator. Refer to previously published papers for more detailed experimental procedures [1,2,5].

### Catalytic pyrolysis and co-pyrolysis using a pyrolysis gas chromatography/mass spectrometry

For more detailed in situ analysis of pyrolysis product composition, a single-shot pyrolyzer (Py-2020iD, Frontier-Lab Co., Koriyama, Fukushima, Japan) connected directly to GC/MS (called hereafter pyrolysis gas chromatography/mass spectrometry (Py-GC/MS)) was used. The pyrolyzer was maintained at 500°C. When pyrolyzing *L. japonica* only, 2 mg of *L. japonica* sample was put in a cup, whereas a mixture of 1 mg of *L. japonica* sample and 1 mg of PP was put in the cup for co-pyrolysis. When the experiments were performed with catalyst, quartz wool was laid over the cup containing the biomass sample forming an intermediate layer, over which 2 mg of catalyst was placed. The pyrolysis product vapor was upgraded catalytically while passing through the catalyst layer. Each test was conducted three times to check the reproducibility. One can refer to a previous paper [1,3] for more detailed experimental procedures.

## Results and discussion

### Characterization of catalyst

The physical properties of Al-SBA-15 are summarized in Table 1. The average pore diameter, total pore volume, specific surface area, and Si/Al ratio were 6.7 nm, 0.9 cm^3^/g, 614 m^2^/g, and 20, respectively.

**Table 1:** Physical properties of catalysts

	**S**_**BET **_**(m**^**2**^**/g)**	**V**_**tot **_**(cm**^**3**^**/g)**	**Average Pore Size (nm)**	**Si/Al ratio**
Al-SBA-15	614	0.9	6.7	20

**Figure 1:**
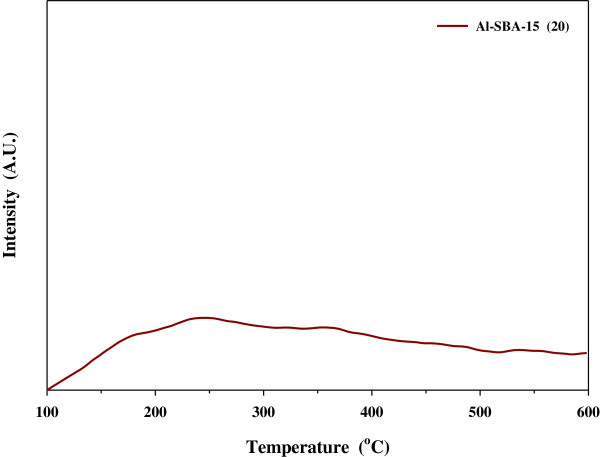
**NH**_**3 **_**TPD of Al-SBA-15.**

Figure 1 shows the NH_3_ TPD analysis results, which represent the acid characteristics of the catalyst. A peak representing weak acid sites was observed at 250°C. XRD patterns of Al-SBA-15 showed good agreement with previously reported results (data not shown), confirming that Al-SBA-15 was synthesized well.

### Catalytic pyrolysis of *L. japonica*

**Figure 2:**
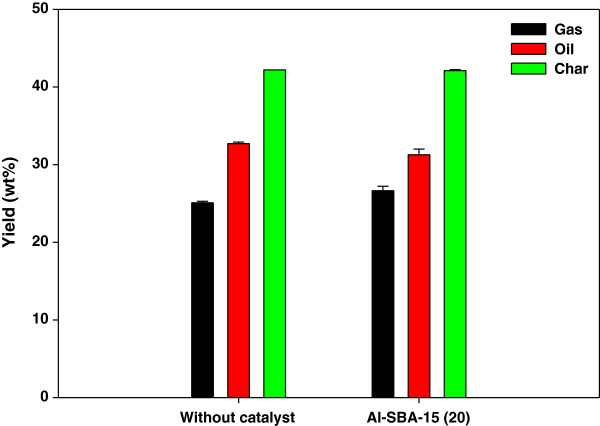
**Product yields of catalytic pyrolysis of *Laminaria japonica.***

Figure 2 shows the results of the catalytic pyrolysis of *L. japonica* performed at 500°C using the fixed-bed reactor. Compared to non-catalytic pyrolysis, catalytic pyrolysis over Al-SBA-15 increased the gas yield from 25.1 to 26.64 wt% and decreased the oil yield from 32.7% to 31.2%. This was attributed to additional cracking and deoxygenation of the vapor products of non-catalytic pyrolysis occurring while they passed through the Al-SBA-15 catalyst layer.

**Figure 3:**
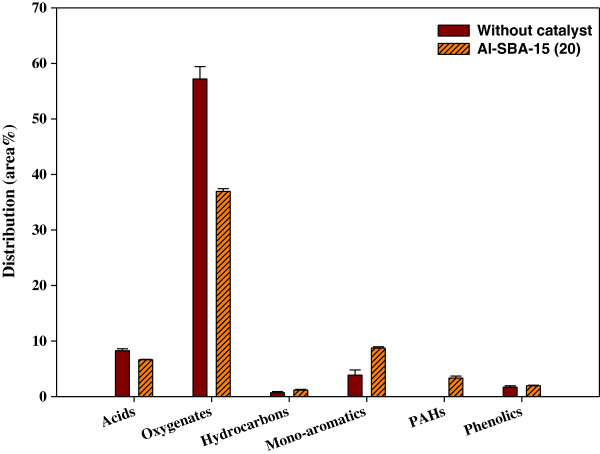
**Product distribution of bio-oil from catalytic pyrolysis of *Laminaria japonica.***

Table 2 shows the gas product species distribution. The contents of CO and C_1_-C_4_ hydrocarbons were increased by catalytic reforming from 2.71 to 3.64 wt% and from 2.61 to 3.97 wt%, respectively. The H_2_O content in bio-oil was increased considerably by catalytic reforming from 42.03 to 50.32 wt%. These results suggest that the most active catalytic reaction of non-catalytic pyrolysis products occurring over Al-SBA-15 with weak acid sites is dehydration, followed by decarbonylation, cracking, and demethylation. Because the average pore size of Al-SBA-15 is relatively high (6.7 nm), large primary pyrolysis product species could diffuse into the pores easily to undergo further reactions, like dehydration, on the weak acid sites of Al-SBA-15.Figure 3 shows the pyrolysis product analysis results obtained using Py-GC/MS. Because pyrolysis bio-oils consist of hundreds of components, they were categorized into seven species groups: acids, oxygenates, furans, hydrocarbons, mono-aromatics, polycyclic aromatic hydrocarbons (PAHs), and phenolics. The analysis result was expressed as peak area percent of each species. The most abundant species found in the non-catalytic pyrolysis product was oxygenates but its content was significantly reduced by catalytic reforming. The acid content was also reduced by catalytic reforming from 8.3% to 6.6%. The reduction of oxygenates and acids by catalytic reforming indicates that oxygen, which causes the instability of bio-oil, was removed significantly from bio-oil, improving its stability. The contents of hydrocarbons and phenolics were not affected much by catalytic reforming. The species whose contents were increased by catalytic reforming were mono-aromatics and PAHs. They were seemingly produced from the conversion of oxygenates and acids on the acid sites of Al-SBA-15.

**Table 2:** **Yield of gas composition from catalytic pyrolysis of *Laminaria japonica***

**Catalyst**		**Without catalyst**	**Al-SBA-15**
Yield (wt%)	CO	2.71	3.64
	CO_2_	19.78	19.03
	C_1_ ~ C_4_	2.61	3.97
Water contents in bio-oil (wt%)		42.03	50.32

**Figure 4:**
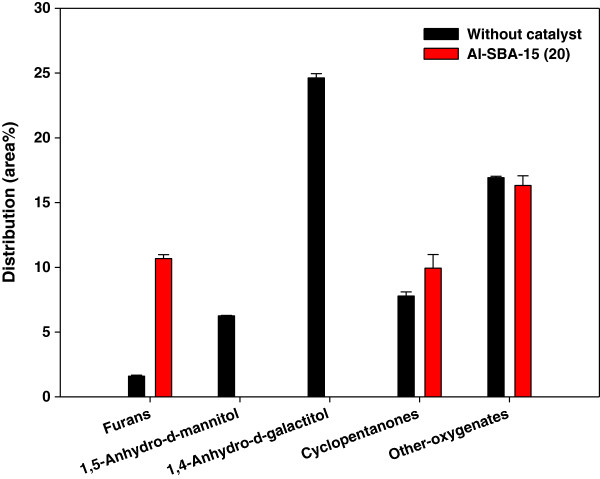
**Detailed species distribution of oxygenates in bio-oil from catalytic pyrolysis of *Laminaria japonica.***

Figure 4 shows the detailed species distribution of oxygenates contained in the bio-oils produced from the non-catalytic and catalytic pyrolysis experiments. 1,4-Anhydro-*d*-galactitol, which was the most abundant oxygenate species (24.6%) in the non-catalytic pyrolysis bio-oil, and 1,5-anhydro-*d*-manitol (6.3%) were completely removed by catalytic reforming over Al-SBA-15. The content of other oxygenates including aldehydes and esters, which also deteriorate the stability of bio-oil, was also reduced significantly by catalytic reforming. Furans can be converted via various chemical reactions to valuable fine chemicals such as medicines, fuel additives, and agricultural chemicals and be applied to the synthesis of polymer materials like polyesters [2]. Therefore, increased production of furans can enhance the economic value of bio-oil. The total content of furans was increased greatly by catalytic reforming over Al-SBA-15 from 1.6% to 10.7%. This was attributed to the conversion of 1,4-anhydro-*d*-galactitol and 1,5-anhydro-*d*-manitol by dehydration and other reactions such as cracking, decarbonylation, etc. occurring over Al-SBA-15 [3]. The content of another high-value-added component cyclopentanone, which can be used for the synthesis of various chemicals including pharmaceuticals and pesticides [18], was also increased by catalytic reforming from 7.8% to 10.0%.

**Figure 5:**
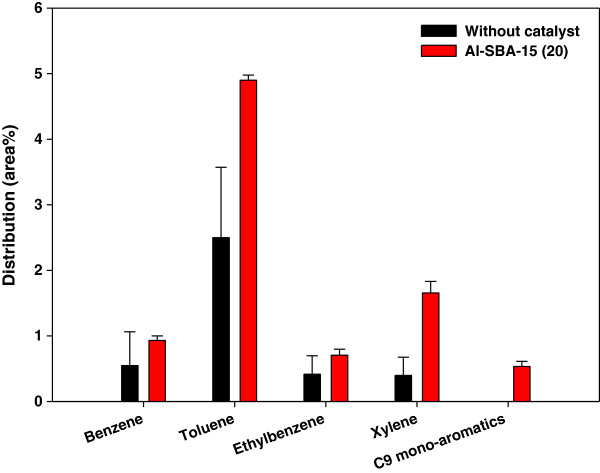
**Detailed species distribution of mono-aromatics in bio-oil from catalytic pyrolysis of *Laminaria japonica.***

Figure 5 shows the detailed species distribution of mono-aromatics, which are often the target high-value-added chemicals of catalytic reforming of bio-oil. The contents of benzene and ethylbenzene were not altered much by catalytic reforming but the contents of toluene and xylene were increased significantly. C_9_ mono-aromatics, which were not found in the non-catalytic pyrolysis bio-oil, were produced from the catalytic reforming. The increased production of mono-aromatics was attributed to the oligomerization and aromatization of pyrolysis reaction intermediates occurring on the acid sites of Al-SBA-15. Previous study [3] has reported that the catalytic pyrolysis of lignocellulosic biomass over Al-SBA-15 produced mono-aromatics via oligomerization and aromatization.

### Catalytic co-pyrolysis of *L. japonica*

**Figure 6:**
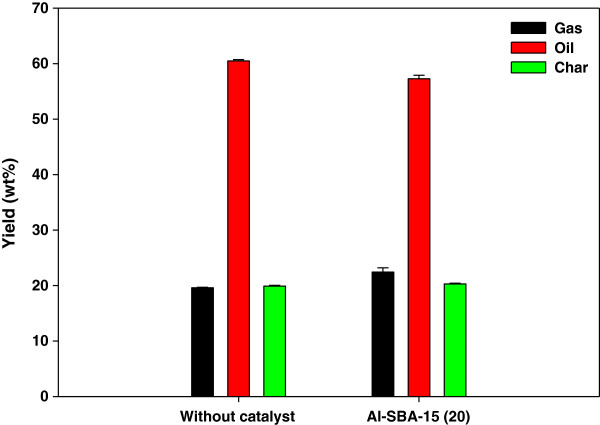
**Product yields of catalytic co-pyrolysis of *Laminaria japonica *** **and polypropylene.**

Figure 6 shows the results of catalytic co-pyrolysis of *L. japonica* and PP using the fixed-bed reactor. Like in the pyrolysis of *L. japonica* only, the catalytic reforming over Al-SBA-15 increased the gas yield and decreased the oil yield. The most dramatic change made by the addition of PP was the reduced H_2_O content in bio-oil. As shown in Table 3, the H_2_O content in the bio-oil obtained from co-pyrolysis was 4.63 wt% (non-catalytic) and 8.93 wt% (catalytic), while that in the bio-oil from the pyrolysis of *L. japonica* only was 42.03 wt% (non-catalytic) and 50.32 wt% (catalytic). The addition of PP enhanced the supply of C and H, resulting in the substantially decreased H_2_O content in bio-oil. Catalytic co-pyrolysis produced more CO, CO_2_, and C_1_-C_4_ hydrocarbons, compared to non-catalytic co-pyrolysis, indicating that deoxygenation reactions were promoted by catalyst. The increase in the water content (from 4.63 to 8.93 wt%) by catalytic reforming suggests the enhancement of dehydration by catalyst.

**Table 3:** **Yield of gas composition from catalytic co-pyrolysis of *Laminaria japonica *** **and polypropylene**

**Catalyst**		**Without catalyst**	**Al-SBA-15**
Yield (wt%)	CO	1.63	2.10
	CO_2_	12.61	13.88
	C_1_ ~ C_4_	5.37	6.46
Water contents in bio-oil (wt%)		4.63	8.93

**Figure 7:**
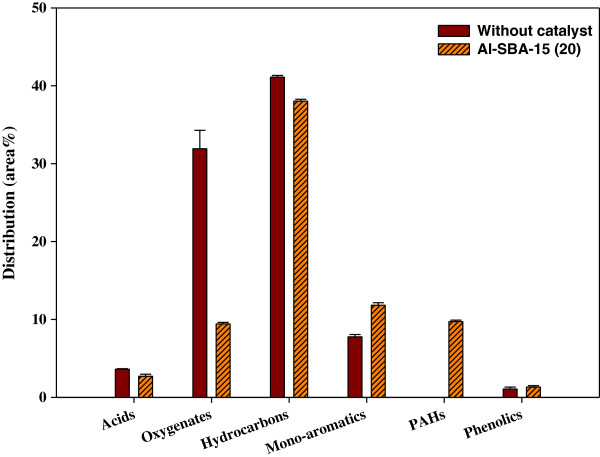
**Product distribution of bio-oil from catalytic co-pyrolysis of *Laminaria japonica *** **and polypropylene.**

Figure 7 shows the species distribution of the bio-oil obtained from the catalytic co-pyrolysis using Py-GC/MS. Compared to the result of the catalytic pyrolysis of *L. japonica* only (Figure 3), the addition of PP increased the content of hydrocarbons enormously, making it the most abundant species in the bio-oil, because the main product species of the cracking of polypropylene are hydrocarbons. Catalytic co-pyrolysis reduced the content of oxygenates considerably compared to non-catalytic co-pyrolysis. This was attributed to the conversion of oxygenates into mono-aromatics or PAHs on the acid sites of Al-SBA-15.

**Figure 8:**
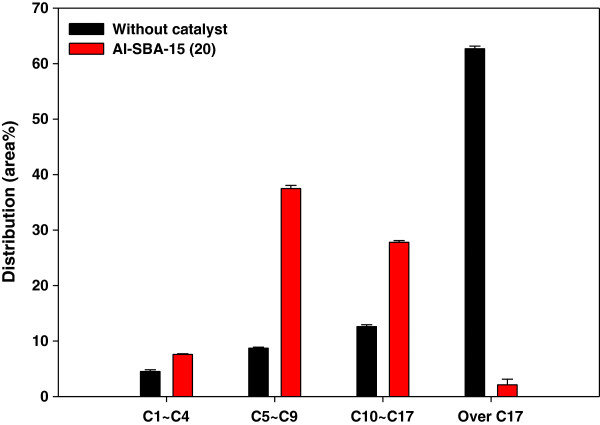
**Carbon number distribution of hydrocarbons from catalytic co-pyrolysis of *Laminaria japonica *** **and polypropylene.**

Total hydrocarbon content was reduced a little by catalytic reforming. According to the carbon number distribution of hydrocarbons shown in Figure 8, non-catalytic co-pyrolysis produced mainly large-molecular-mass hydrocarbons (≥C_17_). These wax species must be decomposed using adequate catalysts because they cause process blockage and deteriorate the oil quality. In this study, most large-molecular-mass hydrocarbons were removed by Al-SBA-15. They are believed to have been cracked into gasoline-range hydrocarbons (C_5_-C_9_) and diesel-range hydrocarbons (C_10_-C_17_) on the acid sites of Al-SBA-15. A previous study on the catalytic pyrolysis of PP over Al-SBA-15 reported that Al-SBA-15 decomposed PP into C_5_-C_17_ hydrocarbons [19].

## Conclusions

The catalytic co-pyrolysis of *L. japonica* and polypropylene resulted in the production of bio-oil with significantly higher quality compared to the catalytic pyrolysis of *L. japonica* only or the non-catalytic co-pyrolysis. The water content in the bio-oil produced from the catalytic co-pyrolysis was 8.93 wt%, which was much lower than that for the catalytic pyrolysis of *L. japonica* only (50.32 wt%). Co-pyrolysis also considerably increased the contents of light hydrocarbons and mono-aromatics that have high economic values. The main hydrocarbon species obtained from the catalytic co-pyrolysis were gasoline-range (C_5_-C_9_) and diesel-range (C_10_-C_17_) species, whereas non-catalytic co-pyrolysis produced mainly wax species (C_17_ or larger). The production of these valuable species was attributed to the catalytic conversion of oxygenates, acids, and heavy hydrocarbons occurring on the acid sites inside the large pores of Al-SBA-15.

## Abbreviations

Py-GC/MS: pyrolysis gas chromatography/mass spectrometry; TPD: temperature-programmed desorption; XRD: X-ray diffraction patterns.

## Competing interests

The authors declare that they have no competing interests.

## Authors’ contributions

HYL, SJC, SHP, JKJ, SCJ, and SCK participated in some of the studies and participated in drafting the manuscript. YKP conceived of the study and participated in all experiments of this study. Also, YKP prepared and approved the final manuscript. All authors read and approved the final manuscript.
